# SOOTHER TRIAL: Observational study of an over-the-counter ointment to heal anal itch

**DOI:** 10.3389/fmed.2022.890883

**Published:** 2022-09-14

**Authors:** Isaac Felemovicius, Robert A. Ganz, Mohammad Saremi, William Christopfel

**Affiliations:** ^1^Voyage Healthcare, North Memorial Hospital, Robbinsdale, MN, United States; ^2^MNGI Digestive Health, Plymouth, MN, United States; ^3^G&S Labs, Inc., Eagan, MN, United States

**Keywords:** pruritus ani, anal itch, rectal itch, perianal itch, itch

## Abstract

**Introduction:**

Pruritus ani, or rectal or anal itch, is a common perianal disorder that affects ~5% of the population of the developed world. Treatments for this disorder are somewhat limited and include conservative non-medical perianal hygiene care, and topical medical treatments including topical steroids, antibacterial and antifungal agents, and topical anesthetic/analgesics such as lidocaine or capsaicin; astringents and vasoconstrictors such as ephedrine can also be used.

**Methods:**

The study was IRB approved. We assessed the efficacy of a novel, composite, over-the-counter, topical lidocaine ointment that included an epidermal barrier and antimicrobial effect along with the typical lidocaine anesthetizing effect, in a single arm, observational, longitudinal, population of 20 ambulatory pruritus ani patients. Patients applied the ointment twice daily, and were studied for 2 weeks; primary outcomes included time to symptom resolution and clinical exam resolution as measured on a 5-point visual analog scale.

**Results:**

Twenty-nine consecutive patients were screened and 20 patients (12 males; 8 females) were enrolled in the study. Ninety percent of patients achieved 100% symptom resolution by 2 weeks, and most were improved within 72 h of initiating treatment; 95% of patients had a normal visual exam by the 2 week endpoint. There were no significant adverse events attributable to the therapy.

**Conclusion:**

Use of a novel composite topical lidocaine agent, demonstrated rapid and effective relief of pruritus ani in an ambulatory population. Additional studies are underway.

**Clinical trial registered:**

Clinicaltrials.gov, identifier NCT05288907.

## Introduction

The medical condition known as pruritus ani (commonly referred to as anal itch) is probably the most common ano-rectal disorder in America, and in the developed world. It has been estimated that ~5% of Americans experience some level of anal itch on a daily basis, so this malady can affect as many as 15 million US residents at any given time (1–3).

Pruritus ani can have multiple causes including diarrhea or frequent liquid stools, multiple loose or soft stools, stool that adheres to the anus and is not entirely cleared post-defecation, leakage of stool from rectal incontinence or frequent passage of gas with some stool leakage, parasites that affect the GI tract, excess anal moisture or perspiration, perianal staph or strep infection, or yeast or candida overgrowth that affects the anal region. Certain diseases or conditions can increase the risk of yeast infections, such as diabetes mellitus, HIV infection or antibiotic usage. Antibiotic use causes alteration of the native intestinal microbiome, potentially leading to yeast overgrowth of the perianal region. Dermatalogical diseases like psoriasis and eczema can also affect the anal region and cause irritation, and systemic diseases such as Crohn's disease, with fistula formation from the small intestine or colon to the skin surrounding the anus can occur allowing leakage of intestinal effluent to the perianal area causing itch. Other comorbid conditions that can contribute to pruritus ani include pinworms, hemorrhoids, anal fissures, and psychogenic causes. Another consideration is that for whatever reason the anal itch initially occurs, a vicious cycle known as the “itch-scratch-itch” cycle can secondarily occur, wherein scratching the itch causes the release of inflammatory chemokines, which secondarily worsens the itch by causing redness, increased itching and dry skin, thereby causing a “rebound” effect (4, 5).

Treatments for pruritus ani are currently limited. The main goal of treatment is to restore the skin in the perianal region to clean, dry, intact, and asymptomatic skin. Repetitively cleaning the region with non-soap warm water then drying the area is the first non-medical treatment that can be tried. If this fails, then steroid ointments can be tried, with or without antifungal or antibiotic additives. Typical antibiotic or antifungal agents contain heavy metals such as zinc oxide or bismuth oxide in varying concentrations. Anesthetic agents like lidocaine or capsaicin can also be tried; lidocaine is commonly available in concentrations from 1 to 5% but capsaicin in therapeutic doses is typically not available commercially. Astringents and vasoconstrictors such as ephedrine can also be used (6).

Lidocaine is a common topical anesthetic agent commercially available in concentrations from 1 to 5%. Lidocaine alters signal conduction in neurons by blocking sodium channels in the neuronal cell membrane thus creating an anesthetic effect (7).

There is a need for newer medical and non-surgical therapies for the treatment of pruritus ani. The ideal therapy would be highly effective at healing itch, with zero to few side effects. This trial assessed a novel, lidocaine-based, composite topical medical therapy for the healing of pruritus ani.

## Methods

### Study rationale and ointment

The objective and rationale of the trial was to evaluate the efficacy of a novel OTC anal itch ointment on the symptomatic improvement of pruritus ani. This study employed a novel FDA-approved, lidocaine-based, composite topical combination ointment for treating and healing pruritus ani (Rectaid; G&S Labs, Eagan, MN). The composite agent is approved for sale and will be commercially available at retail stores in the United States as an OTC product. As noted above, Lidocaine is a common topical anesthetic agent commercially available in concentrations from 1 to 5%. The composite lidocaine therapy is designed to anesthetize the itch and discomfort associated with pruritus ani, decrease anal sensitivity, improve the epidermal permeability barrier, strengthen keratinocytes and also contains protectant and antibacterial properties. This agent, when used topically will help with pruritus ani primary infection and also the secondary effects of the itch-scratch-itch cycle. The study design was a single arm, uncontrolled, case series. Patients were enrolled consecutively, but not randomly.

### Patient population

Any patient male or female, age 18–90, presenting with pruritus ani, in need of treatment, was eligible for the study. Inclusion criteria included presence of pruritus ani (anal itch/discomfort) for at least 2 weeks, and a compatible physical exam. Patients also had to be willing to participate in the study and be capable of understanding the clinical study procedure and be able to give informed consent. Exclusion criteria included inability to understand informed consent, history of inflammatory bowel disease, known venereal disease, or immunodeficiency disease, history of or current anal or perianal abscess, anal or rectal surgery within the past 12 weeks, pregnancy or breastfeeding female, or signs of other rectal diseases such as anorectal fistula, infection, perianal eczema or tumors.

### Protocol

This was a single-arm, longitudinal case series of 20 consecutive subjects with pruritus ani. The setting was a private practice colo-rectal surgery clinic, part of a large multi-specialty clinic and located in a suburb of Minneapolis (Voyage Healthcare, Plymouth, MN). The purpose of the study was to investigate the effect of a novel, composite Lidocaine ointment on the healing of pruritus ani. Patients were recruited between October, 2018 to November, 2019; data collection occurred during the same time period.

Any patient presenting with pruritus ani lasting at least 2 weeks was assessed in the standard manner per usual care.

A standard history was taken including current symptoms, past medical history including diarrhea, constipation, fecal incontinence, antibiotic use, inflammatory bowel disease, previous pregnancies and any previous ano-rectal surgery, social history, and medication usage including use of any laxatives. A detailed physical exam was performed including an ano-rectal exam to assess the pruritus ani. Detailed demographic information was captured from each patient specific to pruritus ani. The patients were consecutive but not randomized.

A visual exam of the ano-rectum before and after therapy was carried out per standard practice including anoscopy, flexible sigmoidoscopy and/or colonoscopy on an as-needed basis.

There is no strict definition of pruritus ani, but in this study the following criteria were used:

a) Two weeks or longer persistent itch in the ano-rectal region;
b) consistent physical exam with the presence of erythema, inflammation and/or breaks in the anoderm.

Once pruritus ani was confirmed informed consent was obtained for the study.

Patients applied the novel pruritus ani ointment, in a prespecified amount (per packaged applicator), twice daily for 1–2 weeks or until complete resolution of symptoms. In addition to the novel treatment, patients were also maintained on standard care for pruritus ani, including, but not limited to, a high-fiber diet, laxatives as needed and appropriate maintenance of the region of the anoderm by keeping the area clean and dry using non-soapy water and appropriate drying. Patients were followed in the clinic on an as needed basis, but were specifically assessed at 1–2 weeks following diagnosis and starting the novel ointment. The study ended for each patient at follow-up visit.

### Efficacy endpoints

#### Primary efficacy endpoint

The primary endpoint was the rate of improvement or resolution of symptoms (itch and discomfort) at 2 weeks. At least 50% symptom improvement and 50% exam improvement, both, were necessary for a successful endpoint. Symptoms and physical exam were graded on a 5 point visual analog scale. Standardized case report forms were used to collect study data. Patients were asked to grade their pruritus ani symptoms of itch and discomfort, with 0 being no symptoms and 5 representing the worst symptoms; a similar 5 point scale was used for physical exam assessment, with 0 representing a normal perianal exam with no visible erythema, inflammation or breaks in the anoderm, and 5 representing the most severe exam. Since the exam was a subjective assessment, a single investigator did all of the pre and post-ointment exams (I.F.).

### Safety

The ingredients used in the study ointment were deemed as safe, or safer, than existing OTC pruritus ani products, since all of the individual ingredients in the novel preparation are currently available and approved for sale in the US. Study products were compounded under the FDA-approved OTC monograph 21 cfr 346 for Anorectal Topical Products and are commercially approved for use anywhere in the United States. There were no anticipated additional risks beyond that of standard topical pruritus ani OTC therapy. As such, safety was not a primary endpoint of the study; nonetheless all adverse effects related to the study were closely monitored and reported. Any adverse effects were summarized by seriousness, severity, relationship to the ointment, and adverse effect type, and were reported to the IRB.

### Study compliance

The study protocol, consent form, case report forms, and all aspects of the conduct of the study were approved and monitored by the Western Institutional Review Board (WIRB; Puyallup, WA). The investigators conducted this study in accordance with all aspects of the protocol, IRB requirements, the Declaration of Helsinki, and the Code of Federal Regulations 21 CFR § 50—Protection of Human Subjects, 21 CFR § 56—and Institutional Review Boards.

### Statistical analysis

Each patient served as their own control for statistical analysis. All analyses were performed *via* the intention-to-treat principle. A two-tailed, paired Student's *t*-test or the Wilcoxon signed rank test were used to assess the discrete variables. (Based on a 75% success rate for at least 50% symptom improvement using the novel ointment, and assuming 30% improvement with standard intervention, 17 patients were required to have a 90% chance of detecting significance at the 5% level, so the study was adequately powered for the outcomes chosen.)

## Results

Twenty-nine consecutive pruritis ani patients were screened and 20 patients were enrolled in the study from 2018 to 2019; there were 12 males and 8 females (Table 1). Of the 9 patients not enrolled in the study, 7 chose not to participate and 2 were deemed to not have pruritus ani; of the 20 enrolled patients all completed the study and there were no dropouts. At initial presentation, mean symptoms of itch and discomfort as graded by the patients on a 5 point visual scale, with 0 being no symptoms and 5 being the worst symptoms, were 4.4; the mean symptom score after treatment was 0.15 (*p* < 0.008). Mean visual exam scores (erythema, inflammation, breaks in the anoderm) pre-treatment were 3.5, dropping to 0.1 2 weeks post-treatment (*p* < 0.008). Eighteen of the 20 patients (90%) achieved 100% improvement in symptoms within 14 days of therapy, most within the first 72 h of therapy, and 19 of the 20 patients (95%) had a normal visual exam by 2 weeks (Tables 2, 3).

**Table 1:** Patient demographics.

**Patient #**	**Age**	**Weight**
1	64	200 lbs.
2	49	148 lbs.
3	46	200 lbs.
4	66	182 lbs.
5	63	165 lbs.
6	50	191 lbs.
7	65	190 lbs.
8	57	142 lbs.
9	61	200 lbs.
10	23	180 lbs.
11	46	150 lbs.
12	76	139 lbs.
13	48	180 lbs.
14	51	130 lbs.
15	68	160 lbs.
16	67	170 lbs.
17	57	200 lbs.
18	64	175 lbs.
19	76	135 lbs.
20	60	189 lbs.

12 males and 8 females.

**Table 2:** Symptom response.

**Patient #**	**Initial symptoms**	**Follow-up symptoms**
1	5	1
2	4	0
3	4	0
4	5	0
5	4	0
6	4	0
7	4	0
8	4	0
9	5	0
10	4	0
11	5	0
12	5	0
13	4	0
14	5	0
15	5	0.
16	5	2
17	5	0
18	4	0
19	3	0
20	4	0

Scale is 0–5 with 5 being the most bothersome.

**Table 3:** Visual exam response.

**Patient #**	**Initial visual exam**	**Follow-up visual exam**
1	3	0
2	2	0
3	5	0
4	4	0
5	4	0
6	3	0
7	4	0
8	4	0
9	3	0
10	4	0
11	5	0
12	4	0
13	3	0
14	4	0
15	3	0
16	4	0
17	2	0
18	3	1
19	4	0
20	2	0

Scale is 0–5 with 0 being normal and 5 being the worst exam.

**Figure 1:**
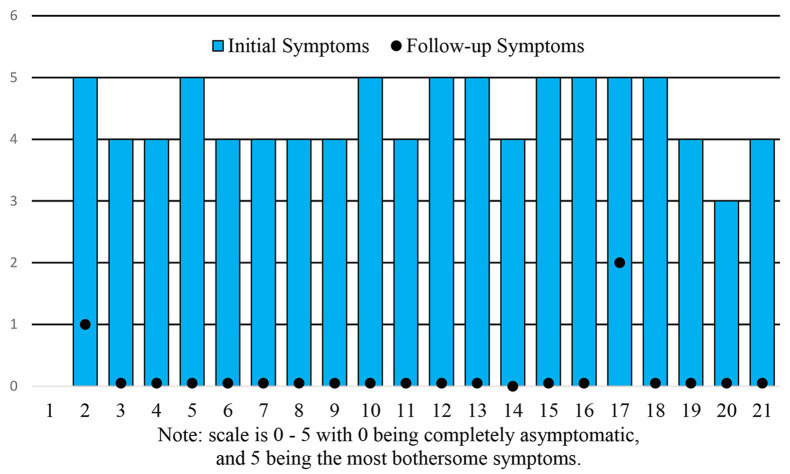
SOOTHER TRIAL results-symptoms. Scale is 0–5 with 0 being completely asymptomatic, and 5 being the most bothersome symptoms.

**Figure 2:**
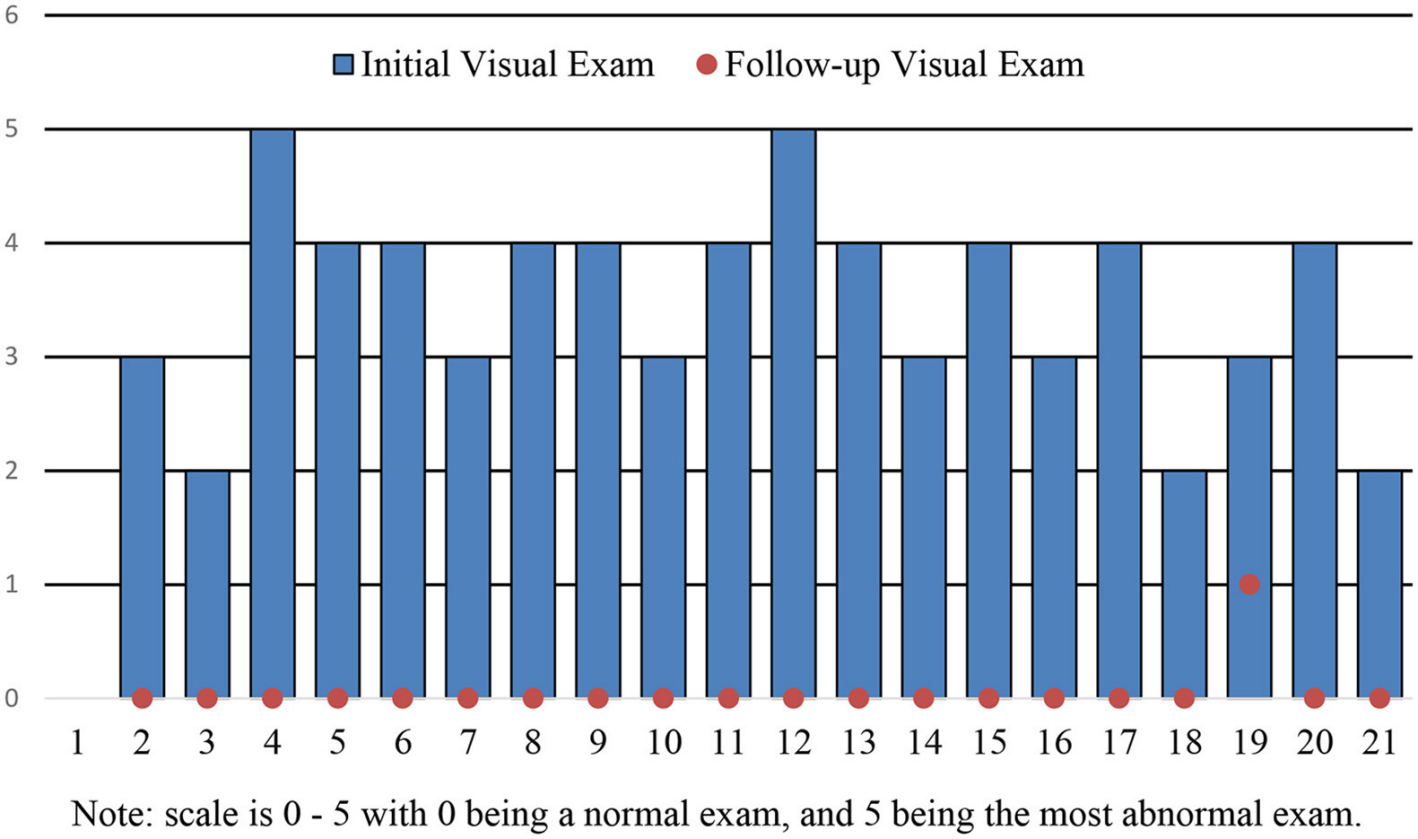
SOOTHER TRIAL results-visual exam. Scale is 0–5 with 0 being normal exam, and 5 being the most abnormal exam.

Per intention-to-treat analysis 100% of patients saw at least a 50% improvement both symptomatically and by exam at 2 weeks (Figures 1, 2). There was one adverse event reported, with one female patient developing hives during the study period although it was deemed unlikely to be related to the pruritus ani product; this patient also had a satisfactory symptomatic and visual response to therapy.

## Discussion

This trial demonstrates an excellent treatment response of pruritus ani to a novel topical composite lidocaine ointment. Ninety percent of clinic patients were asymptomatic by the end of the 2 week study, with most patients achieving a complete treatment response within the first 72 h. The product was very well-tolerated with no direct adverse events. Limitations of this study include a relatively small number of patients, the non-randomized, uncontrolled population, and the potential bias of single physician assessment. Lidocaine is a well-known over-the-counter topical anesthetic agent at commercially available concentrations of 1–5%. Lidocaine is a weak base with a dissociation constant (pKa) of 7.7, and at standard pH (7.4) about half of the molecules are unionized and able to cross into nerve cells, binding to sodium channels inside the cell membrane, preventing nerve depolarization, thus yielding an anesthesia effect (7). Side effects of topical lidocaine are rare but include irritation, erythema or edema of the skin, hives, or tachycardia. The novel ointment used in this study combines the known anesthetic action of standard lidocaine, with keratinocyte and dermal barrier strengthening effect as well as anti-bacterial, anti-fungal properties leading to good results in anal itch.

There have been very few published therapeutic trials in the field of pruritus ani, even though this is a very common disorder affecting up to 1–5% of the general US population (8). Standard treatment consists of good perianal hygiene by keeping the area of the anus clean and dry, treatment of diarrhea, incomplete evacuation and fecal incontinence, use of fiber preparations such as psyllium and oral anti-histamines. Perianal examination to exclude perianal bacterial or fungal infection is necessary and, if indicated, it can be useful to examine the stool for ova and parasites or obtain bacterial cultures. With good compliance, conservative measures can help the majority of anal itch sufferers (4).

Topical agents like 1% hydrocortisone can be tried and in a small, randomized, controlled crossover trial, 68% of patients improved compared to controls. However, use of steroids can result in skin atrophy and fungal overgrowth. Capsaicin, a natural extract of chili peppers, has also been studied in pruritus ani. Capsaicin causes analgesia by activating TRPV1, a permeable calcium ion channel in nerve cells, and depleting substance P a neuropeptide from sensory neurons, which leads to a decreased pain and itch response to local stimuli. A 44 patient randomized, controlled trial of 0.006% capsaicin ointment in a refractory pruritus ani population, resulted in a 31% response rate. Capsaicin does cause a mild perianal burning sensation however. Other agents have also been studied in limited fashion including injection of methylene blue, a neurotoxic agent, and injection of methylene blue in combination with lidocaine and steroid. This type of therapy, however, has been associated with loss of perianal sensation, occasional fecal incontinence and perianal inflammatory reactions (9).

Consequently, there is a need for novel topical agents for the treatment of pruritus ani, in conjunction with conservative measures. In conclusion, use of a novel, topical, composite lidocaine ointment appears to be a promising new agent for the treatment of pruritus ani. Additional studies are pending.

## Data availability statement

The original contributions presented in the study are included in the article/supplementary material, further inquiries can be directed to the corresponding author.

## Ethics statement

The studies involving human participants were reviewed and approved by Western IRB. The patients/participants provided their written informed consent to participate in this study.

## Author contributions

IF helped draft the study protocol, recruited patients, and did all exams. RG conceived the project and helped with study design, drafted the study protocol, helped with data analysis, and drafted the manuscript. MS helped with product design, study design, drafting the protocol, and helped with data analysis. WC helped with product design and study design. All authors contributed to the article and approved the submitted version.

## Funding

This work was supported by G&S, Inc., Eagan, MN.

## Conflict of interest

Authors MS and WC work for Bell International Labs. RG serves as a consultant to G&S Labs, Inc., a subsidiary of Bell International Labs. The remaining author declares that the research was conducted in the absence of any commercial or financial relationships that could be construed as a potential conflict of interest.

## Publisher's note

All claims expressed in this article are solely those of the authors and do not necessarily represent those of their affiliated organizations, or those of the publisher, the editors and the reviewers. Any product that may be evaluated in this article, or claim that may be made by its manufacturer, is not guaranteed or endorsed by the publisher.
